# Knowledge ‘Translation’ as social learning: negotiating the uptake of research-based knowledge in practice

**DOI:** 10.1186/s12909-016-0585-5

**Published:** 2016-02-29

**Authors:** K. L. Salter, A. Kothari

**Affiliations:** Graduate Program, Health and Rehabilitation Sciences, Western University, London, ON Canada; School of Health Studies, Western University, London, Ontario, Canada

**Keywords:** Knowledge translation, Social learning, Reflective practice, Situated learning, Informal knowledge

## Abstract

**Background:**

Knowledge translation and evidence-based practice have relied on research derived from clinical trials, which are considered to be methodologically rigorous. The result is practice recommendations based on a narrow view of evidence. We discuss how, within a practice environment, in fact individuals adopt and apply new evidence derived from multiple sources through ongoing, iterative learning cycles.

**Discussion:**

The discussion is presented in four sections. After elaborating on the multiple forms of evidence used in practice, in section 2 we argue that the practitioner derives contextualized knowledge through reflective practice. Then, in section 3, the focus shifts from the individual to the team with consideration of social learning and theories of practice. In section 4 we discuss the implications of integrative and negotiated knowledge exchange and generation within the practice environment. Namely, how can we promote the use of research within a team-based, contextualized knowledge environment? We suggest support for: 1) collaborative learning environments for active learning and reflection, 2) engaged scholarship approaches so that practice can inform research in a collaborative manner and 3) leveraging authoritative opinion leaders for their clinical expertise during the shared negotiation of knowledge and research. Our approach also points to implications for studying evidence-informed practice: the identification of practice change (as an outcome) ought to be supplemented with understandings of how and when social negotiation processes occur to achieve integrated knowledge.

**Summary:**

This article discusses practice knowledge as dependent on the practice context and on social learning processes, and suggests how research knowledge uptake might be supported from this vantage point.

Over the years, the Evidence-Based Practice (EBP) movement has gained widespread acceptance attributable, in part, to the increasing emphasis on the need for public accountability and transparency in decision-making at the patient, organization and policy levels within the healthcare sector [1, 2]. By shifting the emphasis in decision-making away from clinical expertise and placing it with evidence supplied through rigorous scientific study, practice can be viewed as part of a logical, explicit, transparent, and measurable process [3, 4]. When based on evidence systematically synthesized from methodologically rigorous academic sources, clinical decisions in prescribed circumstances are be viewed as rational, logical and scientific rather than founded on habit or intuition [2, 4]. Over time, practice that is based on the results of methodologically sound research evidence has become synonymous with best practice [3, 5].

Knowledge translation (KT) seeks to narrow the perceived gap between knowledge and practice [6, 7]. KT has been defined as ‘*a dynamic and iterative process that includes synthesis, dissemination, exchange and ethically-sound application of knowledge to improve the health of Canadians, provide more effective health services and products and strengthen the health care system*’ (CIHR, www.cihr-irsc.gc.ca/e/29418.html). To determine which knowledge should be ‘translated’ or promoted for application in practice, KT, like EBP, has relied on the use of the traditional evidence hierarchy in which specific forms of scientific endeavour are assigned greater value on the basis of methodological rigour, while other forms of inquiry and other sources of knowledge or ways of knowing are discounted or rejected [2, 4, 8]. Reliance on linear, techno-rational notions of translation from research generation to selection to creation and dissemination of clinical practice guidelines may have created a ‘second translation gap’ [9]. It has been suggested that it is time to put aside this linear metaphor of knowledge ‘translation’ and contemplate instead the way in which a practitioner might incorporate information from a wider range of knowledge sources in the consideration of practice change [2, 10, 11]. As an alternative, the adoption of research-based evidence by healthcare practitioners can be conceived as part of an ongoing, reflexive and dynamic process of integrated social learning in which knowledge is negotiated, co-produced and emergent [1–3, 8, 12].

If one rejects the assumptions associated with the traditional, linear, EBP-linked view of knowledge translation, is it possible to describe a process of social learning in which knowledge is both emergent, and pluralistic, and in which practice and knowledge are not separated? The purpose of this paper is to discuss how individuals can adopt and apply research-based evidence in practice through ongoing, iterative cycles of learning that include processes of reflection and collective negotiation of shared practices. If the ongoing, learning process through which research-based evidence is selected and integrated by practitioners is better understood, then it may be possible to capitalize on this process by identifying potential points of intervention through which improved research uptake can be facilitated in the future.

We begin, in Section 1.0, by suggesting that, despite the emphasis on a narrowly-defined sample of research-based evidence selected for ‘translation’ into practice settings, multiple forms of evidence are applied at the point of care delivery. Reflective practice, discussed below in section 2.0, is contrasted with evidence-based practice to describe a process that produces contextualized knowledge drawing on a variety of knowledge sources for practice decisions. In section 3.0, the idea of team-based contextualized knowledge developing from individual theories of practice is highlighted. Examples of how social learning communities can negotiate across knowledge sources to derive a communal understanding of practice norms are presented. Implications for practice, related to measuring KT efforts and possible points of intervention to support the uptake of research findings within social learning communities, are discussed in section 4.0.

## Multiple ways of knowing

### Hierarchies of evidence

According to Sackett, the practice of evidence-based medicine represents the conscientious and explicit integration between the best available evidence derived from systematic research with clinical expertise in decision-making [13]. The process of selecting what research findings represent the best available evidence and should be used to inform the development of recommendations, guidelines or protocols and, of course, to guide practice is based on the application of a traditional hierarchy of evidence in which the highest value is assigned to the pooled analysis or systematic review of the results of high-quality, methodologically-rigorous randomized controlled trials (RCTs). The RCT is considered the methodological gold standard for empiric inquiry addressing intervention efficacy [14]. Forms of research inquiry that do not employ research techniques designed to reduce bias and promote internal study validity (e.g., those that offer uncontrolled within or between group observation, report consensus or case study findings) are not considered to be of sufficient rigour to guide practice and can only be considered in the absence of higher quality evidence [2, 4, 15].

Adherence to traditional hierarchy or levels of evidence mechanisms discourages the use of research from a variety of systematic modes of inquiry designated as weak or considered insufficiently empiric [3]. Empiric evidence has been described as observable and reproducible, transparent, open to scrutiny, and originating in experiment and observation rather than in theory [16]. The RCT is an example of a very specific type of empiric research; however, qualitative inquiry also produces empiric results based on observation and is inclusive of both reflection and experience [16]. While it is increasingly recognized that systematic, qualitative study has value in uncovering hidden meaning or exploring knowledge in context, as well as illuminating participant experiences in interventions, the findings, which are not reproducible, are often regarded as not empirical enough [4, 14, 17]. The hierarchies of evidence approaches that currently dominate the EBP-linked KT landscape typically do not recognize or include findings derived from qualitative study.

Research findings are defined, identified, selected, distilled, discussed, and shaped into guidelines that are provided to healthcare practitioners to provide them with direction regarding what to do to achieve a practice standard based on the best available evidence. The use of a hierarchy or levels-of-evidence-based selection process for identifying which research findings are worthy of this type of linear translation results in the selection of a decontextualized and internally valid evidence base that is less well-suited to real world applications than to controlled, experimental environments [2, 3, 18, 19]. The creation of a selected and distilled evidence base will perpetuate the gap between knowledge and practice if the evidence chosen and the knowledge translation tools created from it (e.g., guidelines, recommendations, protocols) conflict with broad-based knowledge user conceptualizations of evidence that are more inclusive of knowledge derived from multiple sources [2, 20]. This includes research conducted using methods not recognized within the dominant evidence hierarchies.

### Practice-based ways of knowing

The notion of evidence as an object to be transferred or translated from researcher to practitioners [1, 8, 21, 22] is a limited conceptualization founded on a series of assumptions about knowledge and practice as follows: a) that knowledge is not emergent, or pluralistic in nature, but instead can be characterized as an objective series of empiric research findings that are value-neutral [4, 10], b) that knowledge can, in fact, be separated from practice in a way that is both useful and meaningful [3, 10, 23], and c) that practice is little more than the application of a series of rapid decisions informed by a selected research evidence base [3, 10].

Practice environments themselves are complex and uncertain, and bear little resemblance to the strictly controlled experimental environment [3]. Context is a complex, multi-layered concept that addresses the practical circumstances of practice environments [14] as well as workplace culture. It is characterised by factors that include organizational artefacts such as institutional language, forms and routines, as well as shared values, team structures and effectiveness, and leadership styles [24]. Information gathered from efficacy research, even when presented in guidelines or recommendations, may not be sufficient to address application in practice environments; healthcare practitioners might judge the information contained in guidelines or recommendations to be impractical or irrelevant, particularly if the ‘evidence’ as presented does not fit with what is already known, based on knowledge gained from multiple sources, including clinical expertise [2, 20, 22, 25]. Further, efforts to stimulate practice changes across an organization may be resisted on account of cultural norms and structural priorities, suggesting that practice improvements need to be supported by organizational structures and processes if they are to take hold. Understanding practice change and the integration of new research findings into applied practice contexts may also require the adoption of a pluralistic definition of evidence more typical of definitions already employed by practitioners [8, 20].

It is generally acknowledged that a variety of knowledge sources inform practice and service delivery. In the context of the real-world practice environment, evidence can be defined, in its broadest terms, as that which informs effective judgement or decision-making [20]. In the description of the practice of evidence-based medicine, we are told that application of ‘best evidence’ must consider clinical experience, and context, including patient circumstance [26]. Indeed, most decisions made in practice are based on information that comes from a variety of sources, including clinical experience or expertise, contextual or cultural knowledge as well as practical, ethical, esthetic, and personal knowledge, rather than the results of selected empiric research alone [3, 4, 20, 27, 28]. Wharf-Higgins and colleagues characterize this information as a composite of ‘evidence’ derived from both hard and soft sources. Soft evidence, the authors suggest, is primarily tacit in nature, built on experience and situated in context, while hard forms are represented by technical and explicit knowledge and information, and that includes research-based evidence [20].

According to Lam [29], human knowledge can be “articulated explicitly” or “manifested implicitly”. The former, explicit (or hard) knowledge is characterised by ease of communication and transfer. Tacit knowledge is, by contrast, personal and embodied. It is, by nature, intuitive, unarticulated and action-oriented [29]. Human activity must be considered in relation to location and cannot be understood effectively in isolation; as humans we engage with both our surroundings and ‘others’ within our surroundings [30]. Learning is generated through integrative interactions with the world and involves both individual and collective practices [29–33]. Practice, Nicolini suggested, is the site of knowing. [30] The way in which soft or implicit, knowledge-in-practice is revealed, and shared between practitioners and across communities plays an important role in how new information is incorporated in the knowledge composite that influences practice [10, 21, 27, 30].

If knowledge-in-practice is emergent, synthesized from different sources within social contexts, the production of more and better clinical guidelines or best practice recommendations is unlikely to narrow the perceived gap between research and practice in a significant way. Research findings, such as those selected for translation via products such as clinical guidelines, represent only one among multiple potential sources of knowledge used in the negotiation of a social and professional understanding of evidence that can be applied in practice [1, 2, 8, 34]. The integration of information based on research findings with knowledge from other sources may be negotiated and applied by healthcare practitioners via processes of individual and collective reflection [1, 35].

There has been an underlying, and perhaps persistent, assumption that evidence-based practice and reflective practice represent two distinct and incompatible frameworks [19]. While EBP and traditional KT have been associated with an “*acontextual, generalized, unbiased and predictive type of knowledge*” (p 132), reflective practice is perceived as being associated with knowledge that is subjective and bound by context [19]. Reflective practice, in addition, is based on the idea that knowledge cannot be meaningfully separated from action [23]. However, these two perspectives both have value when viewed outside of the language of the traditional evidence hierarchy, and placed within the daily practice contexts of healthcare professionals. Understanding the process of evidence integration and self-evaluation of performance in achieving appropriate and acceptable practices is considered part of EBM [26] and reflection is an important means by which one acquires and develops professional knowledge in practice. EBP may benefit from the use of reflective practice, through the valuing and integration of personal and professional judgements and experiences as essential components of the evidence base [19].

## Reflective practice

In 1983, Schön suggested that the dominant epistemology of practice, which he called ‘technical rationality’, was one in which practice consisted of “*instrumental problem-solving made rigorous through application of scientific theory and technique*” (p. 21) [36]. Professional knowledge, suitable for application in instrumental problem-solving was characterized as specialized, firmly-bounded, scientific and standardized. Further, Schön suggested that as an essential step to inform adaptation and adoption, professional knowledge was valued according to an accepted hierarchy in which evidence produced via basic science was assigned greater value than applied science, which in turn was perceived as more valuable than specific problem-solving, skills-based knowledge (Schein, 1973 as cited in [36]). Overall, the further removed from specific or situated context(s) and more general the knowledge or information, the greater the value assigned to it.

Like the creation of a ‘best evidence’ knowledge product or guideline that relies on traditional evidence hierarchies and is separate from, but used to inform action in context, ‘technical rationality’ is an insufficient model to describe clinical practice or practice change [23, 36, 37]. Practice, as Schön observed, is not made up of easily identified and neatly packaged problems with corresponding evidence-based solutions [36]. The scope of practice, the problems and challenges faced by practitioners, are often greater than can be addressed by the direct application of research-based evidence alone. Instead, other forms of knowledge grounded in the experience of practice itself are required, in addition to research-based evidence, in order to address complex, uncertain or ill-defined situations encountered within the practice environment [36–38].

Practices have been defined as combinations of 1) bodily and mental activities, 2) artefacts and their use and 3) a background, or tacit knowledge which both organizes and gives meaning to the practice [39]. Practices are not simply descriptions of human activity, but are also meaning-making, identity-forming and order-producing [30, 40, 41]. Schon suggested that practice referred to coordinated performance of professionals in completion of their tasks as informed by their specific context [36]. Over time, practitioners, or groups of practitioners, negotiate repertoires of techniques, tools, languages, and other explicit artefacts as well as implicit relations, and tacit conventions via interaction with the context and with each other [29, 36, 40].

In the course of daily practice, individual healthcare practitioners develop a repertoire of techniques, expectations, images, perceptions, and so on. Over time, their personal repertoire becomes increasingly spontaneous, automatic and tacit [36]. This experience-based knowledge, or tacit know-how, is generated in the midst of practice [36, 42, 43], sometimes in response to triggers (like unexpected patient outcomes), and is implicit in patterns of action. Reflective practice, or the critical assessment of one’s own actions in order to understand and develop professional skills and abilities [23], provides the practitioner with a means to access this otherwise difficult-to-express form of knowledge and bring experience-based, action-resident knowledge into conscious awareness [3]. Externalization of tacit knowledge, or making the implicit explicit, makes it more amenable to critical review, evaluation, and revision [3, 16, 42].

## Social learning and theories of practice

Knowledge creation or learning within a practice environment is not strictly an individual process. Learning is social, and is as much about social culture, context and lived experience as it is about the acquisition of specific facts or technical skills [33, 40, 43]. While each practitioner within a clinical practice environment constructs her or his own personal theory of practice [23, 36], the development and composition of each personal theory, and the inclusion of both tacit and explicit elements within it, is influenced by social context and culture [22, 43]. As Freeman points out, meaningful action is always informed socially and must, therefore, include an element of interaction [33]. Each personal theory of practice, or behavioural schema is subjected to ongoing examination and adjustment. Practitioners use processes of critical reflection or reflective practice to understand and assess their own actions, to compare and reflect on the experiences of other practitioners and to incorporate credible knowledge from other sources, including valid and reliable research findings, in order to change and improve their personal theories [10, 22, 23, 42, 43].

Externalized tacit knowledge, such as might be surfaced from a process of critical reflection, becomes more mobile and can be shared more easily with others. Individual practitioners might seek out other members of their practice community with whom they can exchange experience-based information as they evaluate and re-evaluate their own evolving theories of practice [20, 22, 43]. The acquisition of experience-based knowledge from other practitioners will also be influenced by the presence (or absence) of shared understandings or perspectives associated with membership in their own local practice culture. Members of the same practice community, for instance, will share overlapping perspectives, and some common tacit understanding for ‘the way things are done’ [34, 42, 43]. Shared narratives or storytelling are commonly used within such a community to share practice-based knowledge [22, 44], as well as knowledge about the local context. Discursive activities such as conversations, discussions, and dialogues are critical tools for establishing associations between different knowledge sources and practice [30]. Interpretation of experiences made available through narrative devices, such as metaphor, used within discursive activity can be facilitated by the common perspectives held within a single practice context or community [40, 42].

Processes of comparison and evaluation are not limited to other team members or professional colleagues who have common contextual understandings. Practitioners also examine their know-how against other available sources of knowledge including formal, research-based evidence [3, 16]. However, the evidence that is considered for this purpose and the extent to which it is integrated into knowledge-for-practice may be influenced by filters derived from personal experience, as well as by other members of the practice environment [43]. A recent scoping review conducted by Thomas & Law [27] identified critical reflection as an important facilitator of research uptake in practice. The review authors suggest that, while tacit knowledge influences the way in which other forms of knowledge are integrated and applied in practice, ongoing reflective practice is an important cognitive process associated with the integration of research evidence.

The process of evaluating and reviewing existing personal practice theory in light of knowledge from other sources and then reconciling and merging new information with it has been described as one of combination [42]. The combination and contextualization of knowledge from varying sources is simultaneously an individual and social process. Combination involves checking and re-checking know-how against the experience of others, examining research-based knowledge and participating in a negotiated process of trial and error to arrive at a revised theory of practice, or a new understanding of how things should be done [22, 40, 42]. This revised behavioural schema can then be internalized by the individual practitioner, filtered through their own tacit knowledge and clinical experience [34, 42, 43]. In this way, practice, or at least the theories of practice or behavioural schemata that are used to guide practice, and the knowledge that informs these theories, is dynamic and emergent.

### Individual and collective mindlines

Gabbay & LeMay theorised that, similar to personal theories of practice, practitioners develop “*clinical mindlines*”, which are internalized, collectively-reinforced, often tacit, and informed by multiple knowledge sources including the practice-based experience of other healthcare practitioners in addition to current clinical practice guidelines, recommendations or practice policy [22]. According to Gabbay & LeMay, mindlines are developed through mechanisms of social learning and, like personal theories of practice or internalized behavioural schemata, they are dynamic and emergent [22]. Mindlines are constantly assessed, evaluated, checked against the experience of colleagues and other sources of information such as clinical practice guidelines or research evidence, tested, and revised [22]. In addition, they may be constrained or enabled by the demands of the clinical organization; that is, the physical, social or cultural context, in which practice occurs [22].

Individual mindlines share a reciprocal relationship with collective mindlines; each contributes to the development of the other [22]. In their extensive ethnographic observations of clinicians within general practices, authors Gabbay & LeMay noted that key to this ongoing and, ultimately successful, process of negotiation between practice knowledge, the evidence, contextual factors and what was actually done was the existence of a thriving community of practice (CoP) that had developed within the observed environment(s) [22].

**Fig. 1:**
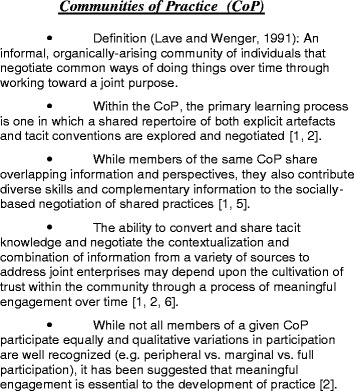
Communities of Practice (CoP)

A community of practice is defined to some extent by engagement in the processes of social practice or creating collectively-negotiated, shared repertoires and shared purpose. Engagement of sufficient density and duration fosters both a sense of community, identity and of belonging [40]. Social practice, or that which takes place in a community, encompasses both explicit (artefacts such as tools, language, role-definitions, and so on) and tacit (conventions, assumptions, values, for example) concepts [40, 45]. The negotiation of collective mindlines by the CoP members provided a shared understanding or consensus that, in fact, represented the accepted norms of appropriate practice for the group. When the results of a collective combination process was internalized by the individual practitioner to become part of their own individual mindline or theory of practice, it would be subject to the influence of their own previous experience and knowledge; however, these personal representations remained within the accepted and agreed upon conventions of shared practice [22]. The social process of collective review, assessment and negotiation has the potential to combine knowledge from formal or research sources with practice-based experience. However, the resulting clinical mindline, theory of practice, or accepted group norm in context may not represent a direct operationalization of the best available empiric research or even the current best practice guideline [20, 22]. What is accepted as ‘normal practice’ may be considered a negotiated truce; the potential for conflict exists amid the vast repertoire of knowledge and perspectives that become apparent when elements of the practice change and conventions are reconsidered [41]. Thus, knowledge-in-practice that has emerged from individual and collective processes of reflection, review, and co-negotiation for application in context might be considered evidence-informed (See Fig. 1 for further information regarding communities of practice).

## Implications

If knowledge ‘translation’ is conceived of as a naturally-occurring, ongoing and iterative process of critical reflection and social learning in which practitioners, both individually and collectively, check, re-check and re-negotiate both personal and shared theories of practice, then the potential to support opportunities for research uptake in concert with this process should be identified.

### Collaborative learning environments

Learning is not constrained to a structured classroom, but is a fundamentally social phenomenon occurring outside of situations one might identify as a formal educational opportunity [40, 43, 46]. In a true learning organization, both informal and formal learning opportunities are respected and encouraged [46]. If the process by which new research-based evidence or evidence-based guidelines are combined with existing knowledge for application in practice is one of social learning [1, 6] then the social context (i.e. the existing practice organization) has the potential to influence this process.

In learning supportive environments, ample opportunity should be provided to facilitate participation in both formal and informal activities associated with learning, including the negotiation of shared repertoire through engagement in discussion, debate or other forms of storytelling, as well as individual engagement in active learning in practice [20, 22, 24, 46, 47]. The CoP is a social, interpretive approach to learning that supports these activities, naturally. Therefore, the formation of spontaneous CoPs should be encouraged and supported [22, 46] and the work of knowledge creation, innovation and skills development legitimized [46]. In so doing, individual practitioners are not viewed as passive subjects to change, but rather as active agents engaged in shaping knowledge to be applied within the practice environment [47].

Active learning is a process of engagement with experience via critical reflection, learning from practice, and evaluation [24]. Activities such as ongoing critical reflection, experiential learning, shared conversation, and collective negotiation help to support evidence-informed change in the workplace [24, 48–50], as do programs that support strong mentorship [51–55]. To address the challenges of practice in complex healthcare environments, practitioners can use the metacognitive processes of reflection to promote change, particularly within contexts described as “*collaborative learning environments*” [27]. A focus on continuous, ongoing learning and improvement encourages reflection on and evaluation of evidence from practice as well as from external sources and their ability to integrate new evidence into practice [48]. The adoption of active learning strategies and support for collaborative learning is not a single person activity -- success in fostering a culture of social, engaged learning depends on the commitment of all team members within the practice environment [24].

### Potential barriers to active and collaborative learning

In healthcare settings, joint or team working can be about creating new ways of thinking and communicating across professional boundaries that includes negotiating shared knowledge and ways of doing things that are accepted in context [56, 57]. Of course, simply linking individuals together in a practice group or team does not instantly create a learning culture or a community of practice. The presence of strong boundaries established along occupational or disciplinary lines is known to constrain knowledge sharing [57, 58]. Co-creation of knowledge and meaning across established professional boundaries, whether in teams or in communities of practice, depends on the cultivation of trusting relationships and accumulation of social capital, both of which require time and effort [59]. In addition, moving toward an effective culture of learning and practice change requires a willingness to address professional divisions around roles and identities. An unwillingness to examine issues raised by boundaries, possible conflicts, roles and existing power differentials within healthcare contexts limits communication and the exchange or movement of knowledge [60, 61].

Facilitating ‘evidence-informed practice’ is often based on a techno-rational, problem-solving approach rather than a social, collaborative and active learning approach. Didactic programs offer training to promote evidence-based skills such as searching the literature or evaluating research. While these skills are not unimportant, the success of active learning approaches relies on the availability of leaders or facilitators with the appropriate skills and abilities to lead and sustain active learning and practice development based on collaborative or social learning perspectives. Key facilitators/leaders should be identified and appropriate training and leadership development opportunities should be provided to enable leaders to develop skills in supporting active learning, critical reflection on their own leadership style and promoting sustainability of a learning culture [49, 62]. Similarly, knowledge brokers act to support the flow of knowledge acting as learning coordinators within their own community or group or functioning as knowledge ambassadors between groups [58].

### Engaged scholarship

In their recent scoping review, Thomas & Law describe several characteristics of collaborative learning environments that support the uptake of research-based evidence and support evidence-based practices [27]. In addition to working environments that support both individual and collaborative reflective practices and the provision of opportunities for mentoring of students, the authors cited commitment to engaged scholarship, primarily through the use of action-research methods, that included academics, healthcare practitioners and students [27]. Engaged scholarship is defined as a collaborative form of inquiry in which all parties contribute to the co-production of a new, contextualised form of knowledge created from the perspectives and skill sets of all invested parties [63]. This type of collaboration rests on the notion that the processes of knowledge production and translation are social and that the academic and practice worlds can create a collaborative space in which collective action can occur [9]. The expectation that accompanies the idea of co-negotiated knowledge or engaged scholarship is that collaborative action in knowledge production is positively associated with knowledge implementation [9]. If practice is allowed to inform research, through the adoption of action research methods that support collaborative engagement at all stages of inquiry, the results would provide a more realistic representation of what works within real world contexts [64–66].

### Importance of clinical experience

Clinical or professional experience, including tacit know-how, is a highly valued, and frequently consulted source of knowledge [20, 22, 27, 67–69]. New knowledge, whether from individual clinical experience or evidence-based clinical practice guidelines, may be checked repeatedly against the professional experience of trusted colleagues, professional authorities or opinion leaders who share an understanding of both practice culture and context and who are able to assess the fit between new knowledge (e.g. clinical practice guidelines) and the current knowledge held by practitioner(s) or by the community of practice [20, 22]. Credibility, in this instance, is not associated with the methodological or technical quality, but with the professional experience of the source who is perceived as a knowledgeable member of the same professional or practice community, and who shares both professional identity and an understanding of context with the information seeker(s) [20, 27]. Information that seems to fit well and that can be confirmed by a trusted knowledge source within a professional CoP is more likely to be included in the collective process of negotiating shared knowledge-in-practice [20, 22, 28].

Within practice environments, trusted authorities or opinion leaders valued because of their clinical experience influence whether (and to what extent) research-based knowledge or knowledge products are used to inform practice [27]. If respected opinion leaders believe that research-based information should be used to inform practice, then this will influence the way in which this type of information is presented for debate, negotiation and combination [27, 28]. Ensuring that professional opinion leaders who are committed and skilled facilitators in local collaborative learning environments are equipped with high-quality evidence that is contextually appropriate represents one way to work within existing social processes of learning in order to facilitate the uptake of valid research-based information.

### In consideration of evaluation

Revised conceptualizations of the ways in which evidence is integrated into practice requires revisions to the ways in which evidence-informed practice is studied and evaluated. In an evidence-informed practice, like the one described by Gabbay and LeMay [22], knowledge from a variety of sources that include multiple forms of research-based evidence and experience-based know-how, is transformed through negotiation and combination. As knowledge translation and social learning reflects collaborative, engaged and participatory processes, evaluation approaches should reflect these basic values. Strategic evaluation should be appropriate to the evaluation of complex interventions and integrate approaches that are both inclusive and participatory [48, 49]. In the interests of ongoing theoretical development in the area of knowledge translation, evaluations should illuminate how and under what circumstances research-based evidence is used and combined with other sources of knowledge in order to identify appropriate outcomes that reflect integrated knowledge and the related social negotiation processes rather than relying on determinations of whether practice change was achieved. In addition, consideration should be given to the possibility for critical appraisal of all forms of knowledge that become part of the review and negotiation process in the creation of shared knowledge-in-practice [22, 67, 70].

### Additional considerations for future research

It has been suggested that ‘reflective capacity’ is an essential characteristic for professional competency insomuch as the ability to engage in reflective practice provides the practitioner with the means to engage with new evidence and knowledge to improve practice [35, 71–73]. Reflection and reflective practice, both individual and collective, has been identified as a potential facilitator of research uptake by practitioners [27], perhaps through reflection on action in terms of how one could perform to improve future practice [10, 73]. However, relatively little is known about the association between the processes of reflective practice, both individual and collective, and the integration of research-based information into negotiated knowledge-in-practice or clinical mindlines [27]. Future efforts should also examine the development of reflective capacity at multiple levels (i.e. individual, team/group and organizational levels) within an expansive learning organization that recognizes and provides time, space and support for both informal and formal learning activities. Future work might also focus on how reflective capacity is different or similar to organizational readiness for evidence-informed practice.

## Summary

Traditional models of knowledge translation have relied heavily on a linear, techno-rational based model in which selections of research-based information are used to inform practice guidelines that are disseminated for application. It is suggested that knowledge used in practice is collaboratively constructed, drawing upon information from a variety of sources -- not just a selection of research-based evidence, as informed by use of a traditional hierarchy of evidence [2]. This collective negotiation of shared practice represents a triangulation of multiple and valued ways of knowing that is inclusive of the both the cultural and contextual complexities of the healthcare environment [8]. Understanding the individual, collective and social processes of learning and practice, supporting collaborative learning environments and undertaking evaluation that reflects the true complexity of the integrative and negotiated knowledge exchange within the practice environment represent important steps forward.
